# Wholes and subparts in visual processing of human agency

**DOI:** 10.1098/rspb.2008.1363

**Published:** 2008-12-02

**Authors:** Peter Neri

**Affiliations:** Institute of Medical Sciences, Aberdeen Medical School, University of AberdeenForesterhill, Aberdeen AB25 2ZD, UK

**Keywords:** biological motion, recognition-by-parts, inversion effect

## Abstract

The human visual system is remarkably sensitive to stimuli conveying actions, for example the fighting action between two agents. A central unresolved question is whether each agent is processed as a whole in one stage, or as subparts (e.g. limbs) that are assembled into an agent at a later stage. We measured the perceptual impact of perturbing an agent either by scrambling individual limbs while leaving the relationship between limbs unaffected or conversely by scrambling the relationship between limbs while leaving individual limbs unaffected. Our measurements differed for the two conditions, providing conclusive evidence against a one-stage model. The results were instead consistent with a two-stage processing pathway: an early bottom-up stage where local motion signals are integrated to reconstruct individual limbs (arms and legs), and a subsequent top-down stage where limbs are combined to represent whole agents.

## 1. Introduction

Theories of object recognition fall between two main strategies: recognition-as-a-whole, whereby the entire object is matched to a flexible internal representation based on invariant properties and/or alignment to stored models, and recognition-by-parts, whereby the object is decomposed into simple and meaningful subparts that are then assembled into an abstract (typically hierarchical) representation of their relationship within the object of interest (Ullman 1996). For the specific example of recognizing a moving human agent and interpreting his/her actions, recognition-as-a-whole may happen by matching the stimulus to a stored database of whole-body silhouettes that represent each pose of a large set of actions (Lange & Lappe 2006), while recognition-by-parts may happen via decomposition of the stimulus into simple elements such as individual body joints or limbs (Marr & Vaina 1982). At present, there is no conclusive experimental evidence to support either strategy.

The main difficulty in approaching this issue experimentally is that most stimulus manipulations affect both recognition strategies, making it difficult to target them separately. For example, human agents are often scrambled for the purpose of measuring human sensitivity to agency. The scrambling schemes that have been used so far are relatively aspecific, typically involving spatial (Cutting 1981; Proffitt & Bertenthal 1990; Grossman & Blake 2001, 2002) or temporal (Proffitt & Bertenthal 1990; Blake *et al.* 2003; Neri *et al.* 2006) disruption of individual joints, a manipulation that affects the structure of the whole body as well as its subparts. In the few instances where scrambling spared individual limbs (Pinto & Shiffrar 1999; Thompson *et al.* 2005), it disrupted the agent to an extent that the nature of the stimulus and the recognition task were modified significantly, making the results hard to interpret in the context of the distinction between the two recognition strategies and their potential contribution to the common task of identifying the agent.

In order to address this issue, we designed a scrambling protocol that allows independent manipulation of the whole body and its subparts (arms and legs) for a motion-tracked sequence that approximates agency more veridically than the stereotyped locomotion patterns used in most previous studies. Central to our experimental design, the same amount of overall disruption was applied with both manipulations, allowing us to make clear-cut predictions for how the experimental measurements should turn out under the recognition-as-a-whole or the recognition-by-parts hypotheses. The results unambiguously rejected the former, and were best interpreted within a two-stage model for the processing of agency. Furthermore, they demonstrate that the drop in human sensitivity caused by inverting the agents upside down (Sumi 1984; Pavlova & Sokolov 2000; Neri *et al.* 2007) is not observed under all conditions as suggested by previous studies, but is dependent upon which stage in the processing hierarchy is probed by the scrambling manipulation.

## 2. Material and methods

### (a) Visual stimuli

We motion captured the *x*–*y*–*t* coordinates of 26 body markers during a 22.7 s sequence of fighting between two martial arts athletes (Neri *et al.* 2006, 2007). There were 13 markers on each actor: one on the head; two shoulders; two elbows; two wrists; two hips; two knees; and two feet. Head markers were discarded, leaving 24 joint trajectories corresponding to four limbs on each agent. Of these, only three limbs (randomly selected) per agent were sampled on any given instance of the stimulus for a total of two agents×3 limbs×3 joints=18 joint trajectories. Balancing the design for the two scrambling conditions (described below) required that the number of joints per limb was equal to the number of limbs, making it necessary to exclude one limb. Each stimulus was constructed by randomly selecting a 1.5 s segment from the sequence. The selected segment was displayed using a limited lifetime sampling technique whereby the 18 trajectories were randomly sampled by nine dots (4.6 arcmin diameter) that lived for only 120 ms (matching the temporal integration window of local motion detectors; Burr 1980), after which they sampled a different trajectory. Dot appearance and disappearance was asynchronous across dots to avoid motion transients from simultaneous transitions of all sampling dots (see Neri *et al.* (1998) and film in the electronic supplementary material; see also fig. 1*i* in Neri *et al.* (2006) for a detailed diagram showing how limited lifetime sampling plays out in the frame-to-frame film sequence). Dots could be randomly bright (74 cd m^−2^) or dark (0 cd m^−2^) on a grey (37 cd m^−2^) background (ensuring that no change in mean luminance ever took place in our experiments) but did not change colour during their lifetime. The trajectories were sized so that their overall centre of mass (across the entire sample) was centred on an Iiyama monitor driven by a VSG graphics card (Cambridge Research Systems), and they did not extend outside a 6.4°×6.4° region. Observers fixated on a central marker at 57 cm distance from the monitor (fixation was only loosely enforced). All were naive except author P.N. (indicated by the rightward-pointing triangle in figures 2 and 3).

### (b) Trial structure and psychophysical task

Each trial consisted of two 1.5 s intervals, one containing the target stimulus and the other containing the non-target stimulus (in random order), separated by a 1 s blank interval. Observers pressed a button to indicate the target interval (temporal two alternative forced choice design) and triggered the next trial after receiving feedback. The target stimulus was generated as described in §2*a*. The non-target stimulus was generated by a similar procedure, but was further subjected to temporal scrambling of the trajectories (see §2*c*). Each trial belonged to one of four types: upright limb scrambling; upright body scrambling; inverted limb scrambling; and inverted body scrambling. Inverted trials were obtained by inverting both target and non-target stimuli upside down with respect to the centre of the display. The four trial types were mixed within the same block for most data collection (four separate staircases were run in parallel); we also collected data where we blocked upright and inverted trials separately (limb and body scrambling were mixed in all experiments). We did not observe significant differences in the thresholds estimated using these two procedures and we therefore combined the data for the analysis presented here. Observers were introduced to the task by showing them a noiseless version of the entire fighting sequence with an unlimited lifetime of the dots. They were then explicitly instructed to indicate which interval resembled more closely a fighting action between two human agents similar to the one they had been shown.

### (c) Scrambling manipulations

**Figure 1:**
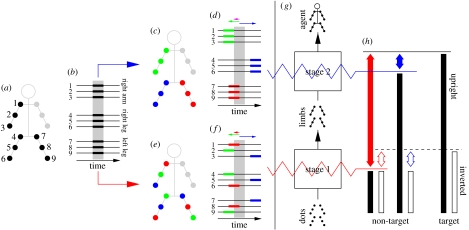
Limb- and body-selective scrambling of joint trajectories. (*a*) Schematic of a point-light agent consisting of 12 body joints and the head (the scrambling procedure is illustrated here for one agent only, but the actual stimulus contained two agents). Only three (randomly chosen) limbs were sampled (indicated by black dots) resulting in nine joint trajectories per agent (the head was never sampled). (*a*,*b*) In the ‘target’ stimulus, the same temporal segment (indicated by grey shading) was sampled for all trajectories (black rectangles are vertically aligned in (*b*). (*c*–*f*) In the ‘non-target’ stimulus, the nine trajectories were grouped into three triplets (one colour per triplet). The sampling segment for each triplet was shifted by a randomly selected amount (indicated by the coloured arrows) resulting in temporal dephasing (the coloured rectangles are misaligned in (*d*,*f*)). No dephasing was present within each triplet (rectangles of the same colour are aligned). (*c*,*d*) In the body-scrambling condition, a given triplet comprised joints coming from the same limb, i.e. each triplet corresponded to one limb (*c*). This triplet assignment resulted in scrambling across limbs (rectangles sampling different limbs are misaligned in (*d*)), but no scrambling within individual limbs (rectangles sampling the same limb are aligned in (*d*)). (*e*,*f*) In the limb-scrambling condition, a given triplet comprised joints coming from different limbs (*e*). For example, the red triplet in (*e*) consists of one joint from the right arm (right shoulder), one joint from the right leg (right foot) and a third joint from the left leg (left knee). This triplet assignment resulted in scrambling within each limb (e.g. misaligned rectangles for the right arm (joints numbered 1–2–3) in (*f*)), but no scrambling across limbs (the same overall temporal region is sampled for each limb in (*f*)). The only distinguishing feature between limb scrambling and body scrambling was the way in which the individual joint trajectories were grouped into triplets; all other sampling manipulations were identical. See the electronic supplementary material film for animated versions of the stimuli. (*g*) Two-stage scheme for a qualitative interpretation of the data. Stage 1 in the processing hierarchy assembles the moving dots into limbs; stage 2 assembles limbs into the full percept of an agent. Limb scrambling (red) places the bottleneck for processing at stage 1; body scrambling (blue) places it at stage 2. (*h*) Consequently, the non-target stimulus only reaches stage 1 on limb-scrambling trials, but is able to reach stage 2 on body-scrambling trials (filled bars above non-target label). The target stimulus (never scrambled) completes stage 2 (filled bar above target label). The perceptual difference between target and non-target is indicated by the filled double-headed arrows, and is larger on limb-scrambling trials (red) compared with body-scrambling trials (blue). Following inversion, which is hypothesized to knock out stage 2, all stimuli are pushed back to stage 1 in the processing hierarchy (open bars). The perceptual difference between target and non-target is decreased by this manipulation on limb-scrambling trials (compare red filled arrow with open double-headed arrow), but remains unaffected on body-scrambling trials (compare blue filled arrow with open double-headed arrow).

In the non-target interval, we scrambled individual trajectories by randomly shifting the sampling segment with respect to the originally selected segment (indicated by the grey rectangle in figure 1) within a temporal window of width *W* centred on the originally selected segment (Neri *et al.* 2006). We express scrambling strength as (*W*−*S*)/*S* (the same definition used in Bülthoff *et al.* (1998) for depth scrambling), where *S* is stimulus duration (width of the grey rectangle). Scrambling could be applied either between (figure 1*c*,*d*) or within (figure 1*e*,*f*) limbs. We describe the procedure for one agent as both agents were similarly manipulated. The nine joint trajectories (3 limbs×3 joints) corresponding to each agent were grouped into three triplets (indicated by three different colours in figure 1*c*–*f*) according to one of two schemes: all joints within a given triplet came from the same limb (body-scrambling condition, figure 1*c*–*d*), or all joints within a given triplet came from different limbs (limb-scrambling condition, figure 1*e*–*f*). For the latter scheme, which joint from which limb was paired with which joint from a different limb was selected randomly (one specific example is shown in figure 1*e*,*f*, but we presented all possible groupings on different trials). After joints were grouped into triplets, joints belonging to the same triplet were scrambled by the same random amount but different triplets were scrambled by different amounts (indicated by the coloured arrows in figure 1*d*,*f*).

### (d) Threshold measurements

**Figure 2:**
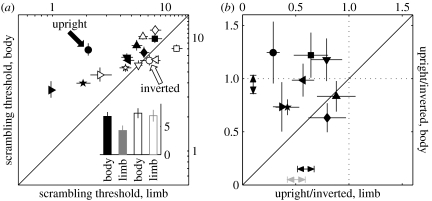
Limb discriminability is better than body discriminability. (*a*) Thresholds for the limb- and body-scrambling conditions are plotted on *x*- and *y*-axes, respectively (see §2 for the definition of scrambling units). Filled symbols refer to upright agents, and lie above the unity line (shown by solid line). Open symbols refer to inverted agents, and fall on the unity line (see main text for statistics). Inset shows average values (±1 s.e.m. across observers) for all four conditions (filled for upright and open for inverted). (*b*) The ratio between upright and inverted thresholds is plotted for both limb- and body-scrambling conditions on *x*- and *y*-axes, respectively. Solid line shows unity line, dotted lines mark unity for individual axes. Vertical double-headed arrow shows observer average (±1 s.e.m. across observers) for the body-scrambling condition, demonstrating that upright/inverted ratios for this condition do not differ from unity (points fall on the horizontal dotted line). Black horizontal double-headed arrow shows observer average for the limb-scrambling condition, demonstrating that upright/inverted ratios for this condition are smaller than unity (points lie to the left of the vertical dotted line) and fall within the range expected from previous studies (indicated by grey horizontal double-headed arrow, which shows the mean±1 s.e.m. of 15 threshold ratios for discriminating the fighting stimuli obtained from Neri *et al*. 2006, 2007). In both (*a*,*b*), error bars show ±1 s.e.m. (not visible when smaller than symbol), and different symbols refer to eight different observers.

**Figure 3:**
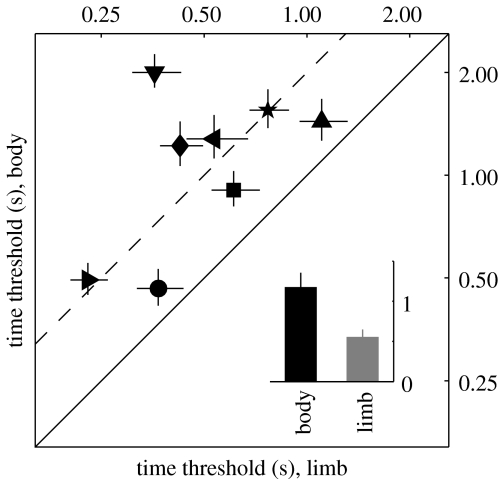
Body processing takes longer than limb processing. Time thresholds (in units of s) for limb- and body-scrambling conditions, plotted on *x*- and *y*-axes, respectively, lie above the unity line (indicated by solid line). The dashed line marks a *y*/*x* ratio of 2. Inset shows average values (±1 s.e.m. across observers) for both conditions.

For measuring scrambling thresholds (figure 2*a*), we determined the percentage of correct target identifications as a function of scrambling strength (using a two-up, one-down staircase) and applied probit analysis to obtain the threshold estimate (Finney 1971). We also analysed the standard deviation of the best-fit cumulative Gaussian (which reflects the slope of the psychometric curve); this was on average one-third of the threshold, and was not statistically different (*p*>0.05 for all paired *t*-tests) between any of the conditions we tested. For measuring time thresholds (figure 3), we selected a large scrambling level and kept it unchanged (this was typically 2–3×the threshold level estimated in figure 2*a*; it was not always possible to use the same multiplicative factor across different observers because for some of them this resulted in scrambling levels that exceeded the range afforded by the stimulus). We then adopted the same procedure and analysis used for scrambling thresholds to determine time thresholds. For time thresholds, the best-fit standard deviation consistently scaled with threshold (i.e. it differed between the two scrambling conditions at *p*<0.01) and was roughly 0.4 of the threshold value. Psychometric curves for both threshold types could be characterized robustly because the experimental design allowed both 50 (chance) and 100 per cent points to be easily obtained in both cases by manipulating the relevant parameter. More specifically, for scrambling thresholds, the 50 per cent point corresponds to zero scrambling strength by definition. As scrambling strength was increased to large levels, the non-target could be easily discriminated from the target by all observers and accuracy reached 100 per cent. Similarly, the scrambling strengths selected for the time threshold experiments were such that, with long temporal exposures, the non-target was easily discriminated from the target at 100 per cent correct performance.

## 3. Results

### (a) Discriminability is better for limb scrambling than body scrambling

Observers were asked to discriminate a target stimulus from a non-target stimulus on every trial (two-alternative forced choice). The target stimulus depicted a fighting sequence between two point-light agents (Neri *et al.* 2006; we used two agents in an effort to preserve the natural structure of the stimulus, but we expect that our results would extend to stimuli containing only one agent). The non-target stimulus was obtained by scrambling the temporal relationship of the dots sampling the fighters (see §2). On different trials, scrambling could be applied either to joints within the same limb while preserving the relationship between limbs (‘limb-scrambling’ condition) or between limbs while preserving the relationship between joints within the same limb (‘body-scrambling’ condition). The two scrambling schemes therefore differed in how disruption was applied across the structure of the agent (figure 1*a*–*f*). However, both scrambling schemes delivered the same amount of overall noise to the observer, rendering the distinction between limb- and body-scrambling trials immaterial to a system that does not represent the limbs as separate perceptual units. For such a system, the unit of representation would be the whole body of the agent, and at the level of the whole body there was no difference in the amount of scrambling between the two conditions (see later in §3 for a demonstration that these considerations are not invalidated by low-level cues, and see §4 for more details).

We measured the threshold amount of scrambling corresponding to correct identification of the target on 75 per cent of the trials. If the human visual system processes agents as perceptual wholes without prior representation of their subparts, then human sensitivity should be affected equally by the two scrambling manipulations and there should be no difference in scrambling thresholds between limb- and body-scrambling conditions. By contrast, if motion signals are initially analysed for the purpose of recovering the structure of individual limbs (stage 1) and limbs are subsequently assembled for the purpose of retrieving whole agents (stage 2), and if these two stages are represented separately in the neural circuitry, we may expect a difference in the threshold measurements.

The results supported the latter hypothesis: scrambling thresholds were better on limb-scrambling trials than on body-scrambling trials by a factor of approximately 2 (filled symbols in figure 2*a* lie above the unity line; paired *t*-test for limb- versus body-scrambling thresholds returns *p*<0.002; when data point for non-naive observer (rightward-pointing triangle) is removed *p*<0.01). This result, which we refer to as the ‘limb-discriminability>body-discriminability’ effect, is consistent with the following two-stage interpretation (figure 1*g*): in the body-scrambling condition, both the target and non-target stimuli reach stage 2 in the processing hierarchy because the limbs are left unperturbed. In the limb-scrambling condition, the non-target stimulus does not progress beyond stage 1 because individual limbs are scrambled (filled bars in figure 1*h*). This leads to a greater perceptual difference between target and non-target in the limb-scrambling condition because the two stimuli are represented at different stages, resulting in better discriminability (filled double-headed arrows in figure 1*h*). This effect is sufficiently strong that it can be demonstrated qualitatively by viewing the stimuli in the electronic supplementary material film, where it can be seen that, for a given scrambling level, limb scrambling degrades the percept of agency more than body scrambling.

### (b) The effect is not due to low-level cues/artefacts

It is possible that, even when the overall amount of scrambling between the two conditions is equated, the two types of scrambling manipulations may involve differences in low-level cues unrelated to agency. For example, it is possible that dots tended to be further apart from each other on limb-scrambling trials as opposed to body-scrambling trials or vice versa (spatio-temporal proximity), and that observers were using this low-level cue (or others such as local rigidity) to perform the task of discriminating target from non-target. In this case, the limb-discriminability>body-discriminability effect would not inform us about the processing of agency, but about uninteresting aspects of our stimuli.

This possibility is very unlikely, because it would predict that the effect should be larger for thresholds that were measured in the presence of large amounts of scrambling, and smaller for thresholds in the lower scrambling range: if scrambling introduces low-level cues that are used by the observer, such cues should become increasingly pronounced as the intensity of scrambling gets larger (indeed, when scrambling=0, there is no distinction between limb- and body-scrambling conditions). In the log–log plot of figure 2*a*, this would predict that data points should move further away from the unity line (larger difference between limb- and body-scrambling thresholds) as they get closer to the upper right corner of the plot (larger scrambling values for the two conditions). This is clearly not the case, in fact the data conformed to the opposite trend (a correlation coefficient of −0.78 between the ratio of the threshold values for the two conditions and their mean; i.e. data points move closer to the unity line as they get closer to the upper right corner of the plot). We conclude that low-level cues are unlikely to be the source of the differential effect we observed for the filled symbols in figure 2*a*. To further corroborate this conclusion, we ran a control experiment.

We inverted the entire stimulus upside down. It is well known that upside down point-light agents are poorly perceived as meaningful human actors (Sumi 1984; Proffitt & Bertenthal 1990; Ahlstrom *et al.* 1997; Pavlova & Sokolov 2000; Tadin *et al.* 2002; Neri *et al.* 2006, 2007), so this manipulation effectively disrupted the percept of agency normally conveyed by this class of stimuli. However, it preserved all low-level structure in the stimulus. If low-level cues resulted in the limb-discriminability>body-discriminability effect, then the effect should not be affected by inversion. If, however, the effect was caused by the percept of agency in the fighting scene, inverting the stimulus should eliminate any differential effect between the two conditions. We repeated our threshold measurements using inverted stimuli and found that the difference between the two conditions was entirely eliminated (open symbols fall on the unity line in figure 2*a*; a paired *t*-test for limb- versus body-scrambling thresholds returns *p*=0.67; when the data point for a non-naive observer is removed *p*=0.85). Combined with the previous observations, these results lead to the conclusion that low-level cues are not the source of the limb-discriminability>body-discriminability effect. More generally, the results from the control experiment demonstrate that this effect is a reflection of the way in which the stimuli are represented by the brain, not of the way in which they were constructed by the experimenter. These results also demonstrate that our scrambling protocol did achieve the desired goal of matching the overall amount of scrambling between the two conditions. Had it not been matched, there is no reason to expect that inversion should lead to limb discriminability=body discriminability.

### (c) Differential effect of inversion on limb discriminability and body discriminability

In going from the main experiment to the control experiment just described (transition between filled and open symbols in figure 2*a*), thresholds for limb- and body-scrambling conditions could have become equal either because the body-scrambling threshold improved, or the limb-scrambling threshold degraded, or a combination of both effects. It is clear from figure 2*a* that only one trend was observed: data points shifted rightwards from filled to open, indicating that limb-scrambling thresholds were degraded by inversion (a paired *t*-test for inverted versus upright returns *p*<0.003), but the body-scrambling thresholds were left unaffected (*p*=0.47). The differential effect of inversion on the two main conditions is demonstrated on a subject-by-subject basis by replotting the data in figure 2*a* as upright/inverted ratios in figure 2*b*. The average ratio for the limb-scrambling condition was approximately 1/2 (see black horizontal double-headed arrow), in excellent agreement with previous measurements of this phenomenon (Neri *et al.* 2006, 2007) (grey horizontal double-headed arrow). By contrast, the average ratio for the body-scrambling condition was approximately 1 (vertical double-headed arrow). Across individual variations, the effect of inversion was significantly more pronounced in the limb scrambling as opposed to the body-scrambling condition (points lie above the unity line in figure 2*b*; a paired *t*-test for body- versus limb-scrambling upright/inverted ratios returns *p*<0.02).

The most parsimonious explanation to account for the entire dataset reported in figure 2 is that inversion disrupts stage 2 more than stage 1. In this scenario, the target and non-target stimuli would be disrupted equally by this manipulation on body-scrambling trials because both stimuli reached stage 2 in the upright configuration for this condition. The perceptual difference between the two would therefore remain similar (compare blue filled with open double-headed arrows in figure 1*h*), leading to no change in discriminability (as we observed experimentally). On limb-scrambling trials, on the other hand, the bottleneck for processing the non-target stimulus is at stage 1, while the target stimulus is still processed by stage 2. Under the proposed scenario, inversion would disrupt the target stimulus more than the non-target stimulus, thus reducing the perceptual difference between the two and leading to poorer discriminability (as we observed; compare red filled with open double-headed arrows in figure 1*h*). This interpretation also predicts that, following inversion, limb discriminability=body discriminability (as we observed), because the inverted non-target is processed mainly by the same stage (1) in both conditions (open bars and double-headed arrows in figure 1*h*). All other possibilities fail to account satisfactorily for our results as they predict either no effect of inversion (if inversion is hypothesized to affect both stages equally or not affect them at all) or better discriminability on limb-scrambling trials following inversion (if inversion is hypothesized to affect stage 1 more than stage 2). We conclude that impairment by inversion, which is commonly used as the higher level signature in studies of biological motion (Sumi 1984; Proffitt & Bertenthal 1990; Ahlstrom *et al.* 1997; Pavlova & Sokolov 2000; Tadin *et al.* 2002; Neri *et al.* 2006, 2007), is mainly due to disruption of processing beyond the limb stage, after the limbs have been recovered but still need to be assembled into an agent. In turn, this indicates that top-down knowledge about the natural orientation of biological motion is fed back more effectively to this stage, and less effectively (or possibly not at all) to pre-limb stages.

### (d) Different time scales for limb discriminability and body discriminability

If the above interpretation is correct and stage 2 depends on top-down feedback more than stage 1, we may expect that processing the agents in the body-scrambling condition (which relies on stage 2) may happen on a longer time scale than the limb-scrambling condition (which relies on a comparison with stage 1), because feedback requires time to be deployed. We tested this prediction by measuring the threshold time required for discriminating target from non-target. Instead of varying the amount of scrambling which was applied to the non-target stimulus as was done for the experiments in figure 2, we kept the amount of scrambling fixed at suprathreshold and varied the duration of the stimuli (see §2). Discrimination in the body-scrambling condition required more than double the time needed in the limb-scrambling condition (points lie above the unity line in figure 3; a paired *t*-test for limb- versus body-scrambling thresholds returns *p*<0.01 (when the data point for a non-naive observer is removed *p*<0.02); mean ratio body scrambling/limb scrambling=2.38), consistent with the feedback interpretation suggested by figure 2*b*. More specifically, the body-scrambling condition required on average an additional 620 ms. This figure may seem large when compared with the reported duration for feedback effects that mediate low-level visual phenomena (Lamme 1995), but is consistent with the extended time scale known to be associated with the processing of complex motion (Neri *et al.* 1998; Poom & Olsson 2002). Given the results in figure 3, it is possible that the effect demonstrated in figure 2 may show some dependence on stimulus duration.

## 4. Discussion

### (a) What is being probed by these experiments?

In the experiments described here, we adopted a 2AFC protocol (which is preferable to a yes–no paradigm; Green & Swets 1966) and observers were explicitly instructed to select the interval containing the stimulus that most closely resembled a fighting action between two human agents (see §2). Is it possible that observers were nevertheless detecting the noise, rather than the agency conveyed by the signal? We can rule out this possibility in our experiments because it is inconsistent with the effect we observed following stimulus inversion (open symbols in figure 2*a*). The inversion effect is widely recognized to provide a signature of the processing of agency (Sumi 1984; Proffitt & Bertenthal 1990; Ahlstrom *et al.* 1997; Pinto & Shiffrar 1999; Pavlova & Sokolov 2000; Tadin *et al.* 2002; Neri *et al.* 2006, 2007); by contrast, there is no logical reason to believe that this effect would be observed for noise. Furthermore, the above-mentioned hypothesis is inconsistent with the significant time difference we observed between limb- and body-scrambling conditions (figure 3). Temporal integration has been shown to extend over long time windows for the processing of agency (Neri *et al.* 1998; Poom & Olsson 2002); by contrast, no evidence suggests that such time scales would apply to a low-level attribute as noise.

A relevant issue partly related to the one above concerns the possibility that the two scrambling protocols may not, in fact, deliver the same amount of noise. Although matched for total power, the two scrambling schemes may implement different degrees of spatio-temporal proximity for correlated noise fluctuations. One simple way to visualize this issue is to think about the different joints as pixels in an array. In the body-scrambling condition, the perturbed pixels are clustered into blocks (limbs); in the limb-scrambling condition, the perturbed pixels are not clustered. This may have led to the differential effect reported in figure 2*a*. However, the above considerations apply equally to the inverted displays: spatio-temporal proximity is preserved by these displays. If the effect reported by the filled symbols in figure 2*a* depended solely on spatio-temporal proximity, this effect should be unaffected by inversion, contrary to what we observed. We can therefore rule out an explanation based on spatio-temporal proximity alone. However, we do not exclude a role for spatio-temporal proximity in the early stages of structure retrieval (our stage 1; see later §4*f*).

### (b) Which specific models are ruled out by the results?

There is currently only one model of biological motion (Lange & Lappe 2006) which has been formulated in sufficient detail to make quantitative prediction for threshold experiments as those performed here. This model would need to be modified in order to account for our results, because it sums energy across all joints via template matching with whole-body silhouettes and would therefore respond equally to limb scrambling and body scrambling. More generally, we can rule out any first-order model that weights different joints independently, whether using the same or different weights across joints. Again, the simple analogy with the pixel array may be useful. A first-order model would correspond to a linear filter applied by template matching with the array. The filter may for example emphasize the feet over other joints (in line with existing experimental evidence for the perception of locomotion; Troje & Westhoff 2006), but does not capture second-order relationships between joints. This model would fail to show the differential effect we observed here because each joint is perturbed by the same amount (on average) in both limb- and body-scrambling conditions. The most direct implication of our results is that joints must be integrated nonlinearly, and that the integration process must be differentially deployed across the body of the agent in a way that reflects a distinction between joints within the same limb and joints belonging to different limbs.

More generally, our results comprise a series of clear-cut empirical effects against which to test future models of subpart processing of agency. Any such future model should not only be able to demonstrate a difference between limb scrambling and body scrambling, but this difference should also be in the specific direction of limb discriminability>body discriminability. Notably, the same model must demonstrate no such difference for inverted stimuli, and most importantly must be able to capture the clear-cut empirical result that inversion only affected thresholds for one condition and not the other (figure 2*b*). Finally, the same model must plausibly account for a time difference in the processing associated with the two conditions of more than 0.5 s. This new set of quantitative observations adds significantly to existing empirical constraints on computational models for visual processing of human agency.

### (c) Representation of limbs as body parts

Previous studies of biological motion have manipulated the trajectories of individual joints (Proffitt & Bertenthal 1990; Blake *et al.* 2003; Neri *et al.* 2006, 2007), and only occasionally the spatial position of the limbs resulting in complete removal of normal body structure (Pinto & Shiffrar 1999; Thompson *et al.* 2005). Neither of these studies perturbed the structure of individual limbs while preserving the relationship among them (as was done here for the limb-scrambling condition), nor did they match the overall level of perturbation across conditions (a feature of our design that was central to the comparison between our two main experimental manipulations). This was also the case for previous studies on the perception of static bodies and isolated limbs (Reed *et al.* 2006), which addressed an altogether different issue. It is clear from these studies that humans can create perceptual representations of individual limbs as well as whole bodies. The central question is whether the representation of whole bodies must rely on prior representation of their parts, or whether the two representations exist separately as wholes. Our experiments demonstrate that, for the purpose of visual detection, the representation of whole agents relies on prior representations of body fragments comprising more than one joint but limited to a limb or similar subpart.

### (d) Specificity of the inversion effect

The results in figure 2 provide the first example, to my knowledge, in the literature of a differential effect of inversion on the same task and target stimulus. A set of previous experiments has demonstrated a differential effect of inversion on static pictures of whole bodies as opposed to static pictures of isolated limbs (Reed *et al.* 2006), but the two sets of stimuli differed so markedly that task and target conditions were also very different, making it difficult to pinpoint which factors determined the differential effect of inversion on the two classes of pictures. In our experiments, these confounds were not present as there was only one difference between the two conditions we studied, namely the type of scrambling manipulation that was applied to the non-target; other than that, observers were performing the same task attempting to identify the same target on all trials and were not aware of any difference in the scrambling protocols between limb- and body-scrambling trials (at the end of data collection, each naive observer was asked whether they had noted any difference in the nature of scrambling for different trials, and none of them reported noticing any difference). In addition, we used moving point-light stimuli rather than static pictures, making our results immediately relevant to action representation.

Because previous studies of inverted biological motion have not focused on the distinction between whole and parts, they have applied scrambling indiscriminately across the agent (Grossman & Blake 2001; Neri *et al.* 2007) or they did not scramble the agent at all (Sumi 1984; Pavlova & Sokolov 2000). These studies exposed an inversion effect but it could only be interpreted in general terms. Our results take a first step in the direction of dissecting this effect, which also occurs for faces (Valentine 1988; Martelli *et al.* 2005), by demonstrating that it is quantitatively accounted for by reference to only a subset of the processing operations that subserve agent identification.

### (e) Predictions for future imaging and electrophysiological experiments

The role of body-part processing has been investigated in previous studies by dismembering the agent via displacement of the limbs apart from their approximate position within the body (Pinto & Shiffrar 1999; Thompson *et al.* 2005) or by presenting limbs in isolation (Bonda *et al.* 1996; Seitz 2002; Reed *et al.* 2006). Both manipulations have been used in the imaging literature, but the resulting data are difficult to interpret in the context of the specific question asked here. When the agent is taken apart, observers adopt entirely different task strategies most likely involving attentional tracking of individual body parts; this interpretation has been used to account for larger activity in response to dismembered agents as opposed to intact ones in parietal areas commonly associated with object tracking (Thompson *et al*. 2005; attentional tracking of individual stimulus items was deliberately minimized for our displays by the use of a limited-lifetime sampling technique (Neri *et al.* 1998), see §2 and the electronic supplementary material film). When body parts are presented in isolation and the resulting brain activity is compared with viewing full bodies, specific brain areas respond to either class of stimuli (Bonda *et al.* 1996). This, however, is not necessarily an indication that full bodies are processed in two stages; rather, it may reflect a specialization for perceiving full bodies as wholes in one area, and a completely separate specialization for perceiving body parts in a different area. Indeed, this interpretation has been used to account for recent results from a series of repetitive transcranial magnetic stimulation experiments involving visual presentation of static body postures (Urgesi *et al.* 2007). Our results predict that if an area exists for the specific purpose of processing whole agents, as seems to be indicated by various imaging studies (see Peelen & Downing (2007) for a recent review), either activity in this area must be dependent upon activity in a separate area where body fragments such as limbs are processed, or limbs must be processed and represented within the same area at a scale that is not resolved by conventional functional magnetic resonance imaging (fMRI) measurements. The former hypothesis may be tested by performing correlational analyses of inter-area activity (Friston 1994), the latter by probing local circuitry using fMR-adaptation paradigms (Krekelberg *et al.* 2006).

As well as predicting that subpart representations would exist within the circuitry that supports whole agent processing, or that such representations would be fed onto this circuitry from a separate module, our results predict that the associated neural processes should occur at different time scales. We observed a difference of more than 0.5 s between limb- and body-scrambling conditions; this time scale may be beyond the temporal resolution of fMRI measurements, but it is well within the range that can be resolved by magnetoencephalographic or visually evoked potential experiments. We predict that this class of electrophysiological measurements would expose two different stages occurring sequentially and separated by approximately 600 ms. The two associated electrophysiological markers should also be highly correlated, in the sense that the occurrence and size of the second event should be conditional upon the characteristics of the earlier one.

### (f) Implications for theories of action processing

Early models of biological motion perception emphasized a bottom-up approach based on invariant structural properties of the skeleton frame and its natural kinematics, such as hierarchical pendular motion (Johansson 1973; Cutting 1981) and rigidity constraints (Hoffman & Flinchbaugh 1982; Webb & Aggarwal 1982). Later models have introduced higher level templates to implement a top-down strategy; the templates are typically derived from a library of whole-body poses and actions (Giese & Poggio 2003; Lange & Lappe 2006). These models have not incorporated explicit subpart representations (although these were implied by variants of recognition-by-parts theoretical frameworks; Marr & Vaina 1982), and lacked the experimental evidence to indicate whether and how they should rely on bottom-up versus top-down processing. Our results are directly relevant to this issue and essentially support a mixed bottom-up top-down model (which shares similarities with Giese & Poggio 2003). Stage 1 implements a structure-retrieval algorithm for the assembly of local motion signals into body fragments, possibly driven mainly by bottom-up information (e.g. spatio-temporal proximity, rigidity). However, the process that subsequently identifies the relevant fragments (e.g. limbs) and assembles them into a whole agent (stage 2) must be of top-down nature if the model is to explain the effect of inversion we report in figure 2. In addition, this process may be generative (Barlow 1985) and may start operating before stage 1 is completed; the two stages would then interact and refine each other in a recursive feed-forward–feedback fashion.

In the context of biological motion, a part-based representation allows for flexible encoding of a large variety of agents and actions using a small number of basic components (Marr & Vaina 1982). This strategy brings obvious advantages to a system with limited neural resources that would otherwise face the curse of dimensionality (Biederman 1987). However, it has been recognized that this strategy works effectively only if the class of objects to be recognized is well captured by the chosen library of part components (Ullman 1996). In the context of agent recognition, it appears that limbs would satisfy this condition as they are clearly essential features of most terrestrial creatures and are very homogeneous across animals: limbs almost invariably conform to the three-jointed structure that is ideal for locomotion (Nagle 1995), and are so fundamental to the body plan of animals that their number can be accurately predicted by remarkably simple equations incorporating only the most basic body structural information (Changizi 2001). Moreover, limbs belong to the class of intermediate complexity features that have been proposed to play a critical role in visual classification (Ullman *et al.* 2002). Our experiments indicate that the human visual system may attach specific significance to this unit of body representation.

## Supplementary Material

Visual stimuli The first two iterations show a 3-s excerpt from the fighting sequence (twice the duration of the stimuli used in the experiments; see Supplementary video 1 in <ce:cross-ref refid="bib25">Neri *et al.* (2006)</ce:cross-ref> for a movie of the entire sequence). In the first iteration all trajectories are visible. The head trajectories (open circles) were not used. For each stimulus, only 3 limbs per agent were sampled; these are indicated by black dots (same convention as in <ce:cross-ref refid="fig1">figure 1</ce:cross-ref>*a*) for the example shown here (different limbs were selected randomly for different stimulus presentations). The resulting 18 trajectories (2 agents×3 limbs×3 joints) were sampled using a limited-lifetime procedure as demonstrated in the second iteration (grey background); this sequence provides an approximation to the actual stimulus that was used in the experiments. The first two iterations just described refer to the ‘target’ stimulus. The third and fourth iterations show the results of applying the limb-scrambling procedure to the same excerpt to obtain a ‘non-target’ stimulus (in the actual experiments target and non-target were generated from different randomly selected excerpts). In the third iteration, joints marked by the same colour undergo an equal temporal phase shift, but different phase shifts were applied to the different colours. The colour grouping is such that joints belonging to the same limb are dephased (scrambled) with respect to each other, but average limb trajectories are in-phase. The fifth and sixth iterations show the results of applying the body-scrambling procedure. Now the colour grouping is such that joints belonging to the same limb are in-phase, but the different limb trajectories are dephased with respect to each other. It can be seen that limb-scrambling (iterations 3–4) reduces the percept of agency in the stimulus much more than body-scrambling (iterations 5–6), even though the same set of phase shifts was applied in both cases. This effect is quantified by the data reported in <ce:cross-ref refid="fig2">figure 2</ce:cross-ref>*a*, where it is also shown that it is not a mere consequence of low-level differences between the two manipulations.
